# Anomalous Systemic Arterial Supply to the Basal Segment of the Lung Treated With Surgery Versus Transcatheter Embolisation: A Report of Two Cases

**DOI:** 10.1002/rcr2.70652

**Published:** 2026-07-19

**Authors:** Hidenori Takahashi, Shinichiro Ota, Yugo Satake, Hiroki Nagamatsu, Ryutaro Hirose, Mio Toyama‐Kosaka, Miwa Morikawa, Takashi Makino, Yuta Tajiri, Natsuko Nakano, Masaharu Shinkai

**Affiliations:** ^1^ Respiratory Disease Center Tokyo Shinagawa Hospital Tokyo Japan; ^2^ Department of Cardiovascular Medicine Tokyo Shinagawa Hospital Tokyo Japan; ^3^ Department of Pathology Tokyo Shinagawa Hospital Tokyo Japan

**Keywords:** anomalous systemic arterial supply to the basal segment of the lung, aspergillosis, cavitary lesion, embolisation, segmentectomy

## Abstract

Anomalous systemic arterial supply to the basal segment of the lungs (ABLL) is a rare congenital vascular anomaly, and its treatment remains disputed. We report two cases of ABLL that were asymptomatic and managed using different strategies based on infection status and anatomical complexity. A 25‐year‐old woman presented with a right lower lobe cavitary lesion supplied by an anomalous coeliac artery branch. Due to suspected chronic fungal infection, thoracoscopic right basal segmentectomy was performed, and subsequent pathology and cultures confirmed chronic cavitary aspergillosis. A 33‐year‐old man presented with an anomalous branch from the descending thoracic aorta supplying the left basal segment. Surgical resection was considered potentially more invasive because of an incomplete fissure and a common left pulmonary vein trunk; therefore, transcatheter embolisation was successfully performed. These cases suggest that treatment selection should be individualised and guided not only by vessel diameter but also by associated infection and anatomical complexity.

## Graphical abstract

Anomalous systemic arterial supply to the basal segment of the lung may require individualised treatment according to associated complications. Of our two asymptomatic patients, the patient shown in the images underwent surgery for suspected chronic fungal infection, whereas the other underwent transcatheter embolisation for non‐infected disease with unfavourable operative anatomy.

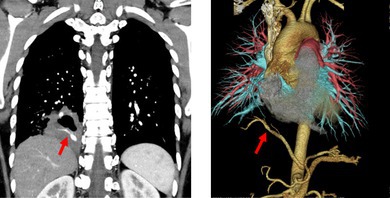

## 1 Introduction

Anomalous systemic arterial supply to the basal segment of the lung (ABLL) is a rare congenital anomaly in which a portion of the otherwise normal lung parenchyma receives blood from an aberrant systemic artery instead of the pulmonary arterial circulation [1, 2]. It most commonly affects the left lower lobe and usually arises from the descending thoracic aorta, although origins from the abdominal aorta, coeliac artery and intercostal arteries have also been reported [2]. In contrast to classical pulmonary sequestration, the involved lung segment has normal bronchial communication and pulmonary venous drainage despite its aberrant systemic arterial supply [3, 4, 5]. Computed tomography (CT) angiography is central to diagnosis and treatment planning, and three‐dimensional reconstruction may further facilitate anatomical assessment [1].

Although patients may be asymptomatic at diagnosis, treatment may be considered because persistent systemic arterial inflow can lead to haemoptysis, pulmonary hypertension, heart failure, infection or rupture of the aberrant artery [3, 4, 5]. Traditionally, surgical resection has been performed; however, endovascular embolisation has emerged as a less invasive alternative in selected cases [1, 5]. We report two asymptomatic patients who underwent different treatments, surgery and embolisation, based on infection risk and anatomical complexity, respectively. These two cases illustrate practical decision‐making between surgery and embolisation.

## 2 Case Report

### 2.1 Case 1

**FIGURE 1:**
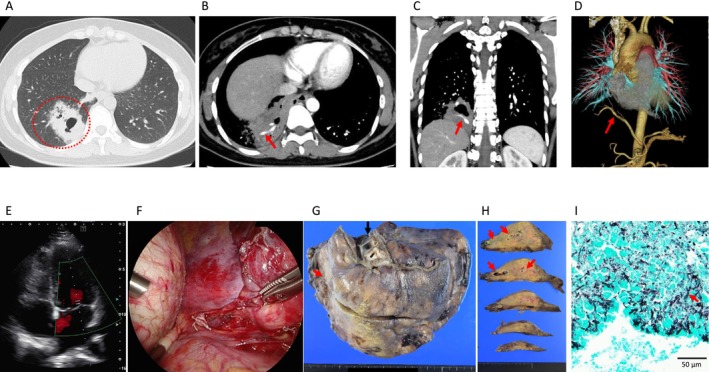
(A) Chest computed tomography (CT) showing a cavitary lesion in the right lower lobe near the diaphragm (red circle); (B, C) contrast‐enhanced CT showing an anomalous vessel traversing the cavitary lesion on axial and coronal images (red arrows); (D) three‐dimensional CT angiographic reconstruction showing an anomalous systemic artery arising from a branch of the coeliac artery (red arrow); (E) Colour Doppler transthoracic echocardiography demonstrating an eccentric aortic regurgitation jet; (F) intraoperative thoracoscopic view showing the anomalous systemic artery during surgical dissection and division; (G, H) gross specimen of the resected right basal segment, with the external view showing the bronchial and pulmonary vascular stumps (black arrow) and the anomalous systemic artery (red arrow), and serial cut sections showing a yellow‐grey poorly aerated lesion within the basal segment (red arrows); (I) Grocott staining demonstrating fungal hyphae morphologically consistent with *Aspergillus* spp. (red arrow). Scale bar = 50 μm.

A 25‐year‐old woman was referred to our hospital after an incidental right lower lobe abnormal opacity was detected on chest radiograph during a routine health check‐up. She was asymptomatic, had no history of tuberculosis or immunosuppression and was a nonsmoker. At presentation, the patient was afebrile with stable vital signs and no respiratory distress. The respiratory and cardiovascular examinations were unremarkable. CT revealed a right lower lobe cavitary lesion, near the diaphragm (Figure 1A). Contrast‐enhanced CT showed an anomalous vessel traversing the lesion on axial and coronal images (Figure 1B,C). Three‐dimensional CT angiographic reconstruction further revealed that this anomalous systemic artery arose from the coeliac artery, passed through the diaphragm and supplied the right basal segment (Figure 1D). The maximum vessel diameter was 9.8 mm.

Laboratory tests showed no elevation in C‐reactive protein levels, and routine blood tests were within normal limits. MAC antibody, T‐SPOT.TB, myeloperoxidase‐antineutrophil cytoplasmic antibody and PR3‐ANCA were negative. In contrast, the serum β‐D‐glucan level was elevated at 44.3 pg/mL, and the serum aspergillus galactomannan antigen test was positive. Because the lesion was cavitary, a comprehensive diagnostic evaluation of infectious aetiologies was necessary, particularly to exclude pulmonary tuberculosis. Bronchoscopy and bronchial lavage were performed. However, bacterial, mycobacterial and fungal studies of the lavage fluid were negative, and mycobacterial PCR was negative. Transthoracic echocardiography revealed normal left ventricular systolic function, bicuspid aortic valve with suspected fusion of the right and left coronary cusps and trivial aortic regurgitation with a posteriorly directed eccentric jet (Figure 1E).

Despite the negative bronchoscopic microbiological results, infection within the involved lung segment could not be ruled out because of the imaging findings and positive fungal markers. After multidisciplinary discussions with the thoracic surgery and cardiology teams, pulmonary abscesses and fungal infections were considered as the working diagnoses. A catheter‐based approach was not the favoured initial method because surgical resection was thought to provide definitive treatment for both the anomalous artery and the suspected infected cavitary lesion. Therefore, preoperative therapy with oral amoxicillin‐clavulanate plus itraconazole was administered for 7 weeks, followed by thoracoscopic right basal segmentectomy. Intraoperatively, mild inflammatory fibrous adhesions were observed at the lung base. The anomalous systemic artery supplying the right basal segments was identified, ligated and divided, followed by the division of the basal pulmonary vessels and bronchus (Figure 1F). The intersegmental plane was determined using the inflation–deflation method. A gross examination of the resected specimen clearly demonstrated an anomalous systemic artery supplying the right basal segment (Figure 1G,H). Histopathological examination confirmed an aberrant systemic artery entering the basal segment along with cystically dilated bronchi and chronic active inflammatory changes. Grocott staining demonstrated fungal hyphae consistent with *Aspergillus* spp. (Figure 1I), and a culture of the surgical specimen yielded *Aspergillus fumigatus*. There was no evidence of malignancy. The definitive diagnosis was anomalous systemic arterial supply to the right basal segment complicated by chronic cavitary aspergillosis. The postoperative course was uneventful. No evidence of recurrence was observed on serial imaging during the first three postoperative months, and itraconazole therapy, which had been initiated preoperatively, was continued as maintenance therapy.

### 2.2 Case 2

**FIGURE 2:**
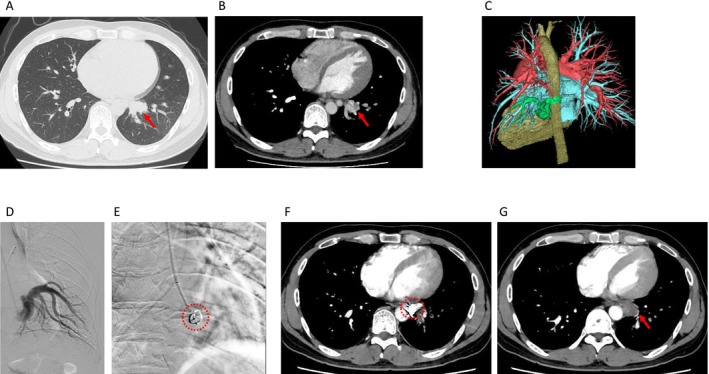
(A, B) Computed tomography (CT) images showing a nodular, mass‐like lesion in the left lower lobe, which corresponded to the anomalous systemic artery (red arrow). (C) Posterior oblique three‐dimensional CT angiographic reconstruction showing an anomalous systemic artery (green) arising from the descending thoracic aorta and supplying the left basal segment. (D, E) Angiographic findings during transcatheter embolisation: Selective angiography showing the anomalous systemic artery and its distal branches (D), and completion angiography confirming occlusion of the anomalous artery, with the embolisation device indicated by the red circle (E). (F, G) Follow‐up contrast‐enhanced CT obtained 1 week after the procedure. (F) Stable position of the embolisation device without migration (red circle). (G) No contrast enhancement of the anomalous artery (red arrow), consistent with thrombosis.

A 33‐year‐old man was referred to our hospital after an incidental nodular lesion was detected in the left lower lobe on CT (Figure 2A,B). The patient was asymptomatic, had never smoked, and had no abnormal laboratory findings, including tumour or inflammatory markers. Positron emission tomography‐CT revealed no significant uptake by the lesion.

Three‐dimensional CT angiographic reconstruction revealed an anomalous systemic artery arising from the descending thoracic aorta and supplying the left basal segment (Figure 2C). The maximum vessel diameter was 16.5 mm. CT also showed an incomplete fissure between the lingular segment and the left lower lobe, as well as a common left pulmonary vein trunk. Bronchoscopy revealed arterial pulsation in the left lower lobe. Transthoracic echocardiography revealed mild left atrial dilation with an estimated left atrial volume of 70 mL, which may reflect the subtle haemodynamic effects of the shunt.

Considering the relatively large vessel diameter, catheter‐based embolisation and surgical resection were considered. However, after multidisciplinary discussions involving pulmonologists, cardiologists and thoracic surgeons, primary transcatheter embolisation was selected as a less invasive, lung‐preserving strategy to control the aberrant systemic arterial inflow, because there was no evidence of infection, no urgent need to remove the lung parenchyma, and CT demonstrated anatomical features that could make surgery more complex, including an incomplete fissure and a common left pulmonary vein trunk. The procedure was successfully performed using a 12‐mm Amplatzer Vascular Plug I with additional coil packing. Because selective catheterisation via the femoral approach was difficult owing to the marked angulation of the anomalous vessel, the approach was changed to the left brachial artery. Final angiography confirmed satisfactory occlusion (Figure 2D,E). Simultaneous haemodynamic assessment demonstrated a normal pulmonary artery pressure of 21/8 mmHg (mean, 13 mmHg), pulmonary capillary wedge pressure of 8 mmHg and right atrial pressure of 5 mmHg. Contrast‐enhanced CT performed 1 week after the procedure confirmed thrombotic occlusion of the anomalous artery and a stable device position without migration (Figure 2F,G). Similarly, follow‐up CT performed at 20 months showed no device migration.

## 3 Discussion

We report two asymptomatic patients with ABLL who were managed using different therapeutic strategies. This variability in management has also been reflected in the literature. A recent literature review of approximately 87 reported cases noted that both the necessity of treatment and the optimal therapeutic strategy remain controversial. Whilst embolisation has increasingly been used in recent years, accounting for 74% of treated cases reported between 2005 and 2022 [5], these observations do not establish embolisation as superior to surgery or surveillance, but support its consideration as a less invasive option in carefully selected patients. Our cases further highlight that treatment selection should be guided by infection status, vascular anatomy, haemodynamic burden and the expected invasiveness of the intervention, rather than by vessel diameter alone.

Case 1 illustrates the importance of recognising infections as a major factor in treatment selection. Although the affected lung parenchyma is generally preserved in this anomaly, chronic systemic arterial inflow may cause vascular and parenchymal changes, including thickening of anomalous systemic arteries and pulmonary veins, local congestion and impaired bronchial clearance, thereby predisposing the involved segment to recurrent respiratory infection [5]. Previous reports have described pathogens such as *Staphylococcus*, anaerobic bacteria and *Aspergillus* [5, 6]. In our patient, the cavitary lesion and positive fungal markers raised a strong suspicion of chronic fungal infection within the involved segment, which was subsequently confirmed by the isolation of *Aspergillus fumigatus* from the resected specimen. In this context, surgery was considered preferable because it enabled the removal of the infected cavitary lesion and control of the aberrant artery. The coexistence of a bicuspid aortic valve with an eccentric aortic regurgitation jet may also have contributed to altered local haemodynamics, although this remains speculative [7].

In contrast, case 2 illustrates the potential role of primary transcatheter embolisation in a non‐infected patient with an unfavourable operative anatomy. Although the aberrant artery was relatively large, the incomplete fissure and common left pulmonary vein trunk raised concerns that surgical resection could be more technically complex and potentially more invasive than standard thoracoscopic resection. In the absence of infection and with no urgent need to remove the lung parenchyma, embolisation with an Amplatzer vascular plug and coils was selected as a less invasive alternative and was completed successfully with stable occlusion during follow‐up. This case suggests that anatomical complexity and the need for parenchymal resection, rather than vessel diameter alone, should be considered when selecting the optimal treatment strategy [1].

Collectively, these cases suggest that treatment planning for this anomaly should consider not only the vascular anatomy but also the infection status, haemodynamic burden, potential future complications and expected invasiveness of each intervention. Given the paucity of comparative long‐term outcome data after either surgery or embolisation, careful follow‐up is required to assess late complications and the durability of the selected treatment [5]. Early multidisciplinary collaboration amongst pulmonologists, interventional specialists and thoracic surgeons is important in selecting the most appropriate strategy for each patient.

## Author Contributions

Conceptualisation: H.T. and M.M. Investigation, data curation, formal analysis and writing of the original draft: H.T. Visualisation: H.T. and N.N. Writing, review and editing: S.O., Y.S., H.N., R.H., M.T.‐K., M.M., T.M., Y.T., N.N., M.S. Supervision: M.S. All authors contributed significantly to data interpretation, critically revised the manuscript for important intellectual content, approved the final version of the manuscript and agreed to be accountable for all aspects of this study.

## Funding

The authors have nothing to report.

## Ethics Statement

All procedures were performed in compliance with relevant laws and institutional guidelines.

## Consent

The authors declare that written informed consent was obtained from both patients for the publication of this manuscript and the accompanying images and attest that the consent forms comply with the journal requirements, as outlined in the author guidelines.

## Conflicts of Interest

The authors declare no conflicts of interest.

## Data Availability

The data that support the findings of this study are available on request from the corresponding author. The data are not publicly available due to privacy or ethical restrictions.
